# A Sealed Irrigation Approach for Root Resorption: Case Report With Review of Irrigation Strategies

**DOI:** 10.1155/crid/2066169

**Published:** 2026-02-12

**Authors:** Mohsen Aminsobhani, Nasim Hashemi, Fatemeh Malekpour

**Affiliations:** ^1^ Department of Endodontics, School of Dentistry, Tehran University of Medical Sciences, Tehran, Iran, tums.ac.ir; ^2^ School of Dentistry, AJA University of Medical Sciences, Tehran, Iran, ajaums.ac.ir; ^3^ Department of Endodontics, School of Dentistry, Zanjan University of Medical Sciences, Zanjan, Iran, zums.ac.ir

**Keywords:** endodontics, irrigation, root canal therapy, root resorption

## Abstract

**Background:**

Root resorptive defects present significant diagnostic and clinical challenges, particularly when differentiating internal root resorption (IRR) from external cervical resorption (ECR). Their irregular anatomy complicates effective debridement and increases the risk of irrigant extrusion during conventional irrigation procedures.

**Case Presentation:**

A 33‐year‐old female with a history of asthma presented with a chief complaint related to maxillary lateral incisor #7. Clinical examination revealed normal probing depths, absence of tenderness to percussion and palpation, no mobility, and no response to cold testing. Periapical radiography demonstrated two well‐defined, balloon‐shaped radiolucencies in the coronal third of the canal. CBCT revealed an extensive resorptive defect with perforation of the buccal canal wall, round internal morphology, and thinning of dentinal walls—features most consistent with ECR, although a combined IRR/ECR process could not be excluded. Because of the patient′s sensitivity to the odor of sodium hypochlorite, a sealed irrigation system was employed. A dual‐needle setup was used in which 5.25% NaOCl was delivered apically through a 27‐gauge needle while simultaneous coronal suction via a 22‐gauge needle created controlled negative pressure within a sealed chamber. This configuration allowed continuous irrigant exchange and effective debridement while minimizing extrusion risk and patient discomfort. The canal was obturated using an MTA‐based sealer. At the 17‐month follow‐up, the tooth remained functional and asymptomatic, with radiographs showing periapical stability and no evidence of recurrent or progressive resorption.

**Conclusion:**

This case demonstrates that a sealed, negative‐pressure irrigation technique can provide a safe and effective method for managing complex resorptive defects—particularly when patient‐related limitations and diagnostic ambiguity make traditional irrigation approaches insufficient.

## 1. Introduction

Resorptive lesions of the root are among the most difficult conditions to diagnose and manage in endodontics. One of the greatest challenges is distinguishing internal root resorption (IRR) from external cervical resorption (ECR). Their clinical and radiographic features often overlap, and even advanced imaging such as CBCT may not always provide a definitive diagnosis. Indeed, the recent ESE position statement highlights that in certain clinical scenarios, establishing a clear distinction is not only difficult but occasionally impossible [1, 2].

IRR is generally defined as an odontoclastic process originating within the pulp space, frequently associated with chronic pulpal inflammation, trauma, orthodontic forces, or iatrogenic injury. ECR, by contrast, begins on the external cervical root surface and maintains communication with the periodontal ligament. The etiological factors proposed for ECR include trauma, orthodontic movement, internal bleaching, periodontal surgery, bruxism, and systemic inflammatory conditions. Although the biological mechanisms differ, both forms may present with irregular, invasive defects that complicate cleaning and shaping procedures [3].

Treatment outcomes also vary depending on the type and progression of resorption. IRR confined within the canal space generally has a favorable prognosis when thoroughly debrided and obturated, whereas extensive perforating defects or rapidly progressive lesions are associated with poorer outcomes. Similarly, early‐stage ECR lesions may respond well to nonsurgical management, but advanced defects involving deep penetration into dentin or pulpal communication often require surgical intervention, and in severe cases extraction may be unavoidable [4].

Sodium hypochlorite (NaOCl) remains the irrigant of choice because of its potent antimicrobial activity and ability to dissolve organic tissue [5]. However, traditional syringe‐based positive‐pressure irrigation may not provide sufficient delivery of irrigants into the complex and irregular morphology of resorptive defects. Inadequate penetration of NaOCl permits the persistence of debris, bacteria, and resorptive tissue, compromising treatment outcomes, whereas excessive pressure may increase the risk of irrigant extrusion, postoperative pain, or periapical inflammation [6].

To overcome these limitations, several advanced irrigation strategies have been introduced, including passive ultrasonic irrigation (PUI) [7], the self‐adjusting file (SAF) [8], EndoVac [9] and the Endo Activator [10].

These systems are aimed at enhancing irrigant distribution and activation within anatomically challenging regions; however, no single technique has demonstrated consistently predictable results for IRR or ECR lesions.

This report describes the management of a resorptive lesion exhibiting overlapping features of IRR and ECR, illustrating the diagnostic complexity of such cases. More importantly, it presents a sealed negative‐pressure irrigation technique designed to improve irrigant delivery, safety, and debridement efficiency within complex resorptive anatomy.

## 2. Case Report

Informed consent was obtained from the patient regarding the use of her clinical photographs and radiographs for inclusion in scientific publications and presentations, with assurance that her identity would remain undisclosed.

A 33‐year‐old female patient with a history of asthma and persistent cough was referred with a complaint regarding Tooth #7 (Figure 1a). The patient denied any history of trauma or orthodontic treatment and reported sensitivity to the odor of sodium hypochlorite. Clinical examination revealed an esthetic composite veneer placed approximately 2 years earlier (Figure 1b). Periodontal probing depths on the anterior teeth were within normal limits, with no bleeding on probing. Tooth #7 was not tender to percussion or palpation, exhibited no mobility, and showed no response to pulp sensibility testing. Cold testing was performed using a refrigerant spray (Endo‐Frost, Roeko, Germany) applied to an insulated cotton pellet; adjacent teeth were used as controls. Electric pulp testing (EPT) was performed using a digital pulp tester (Parkell Digitest II, Edgewood, NY, United States), and Tooth #7 demonstrated no reproducible response, confirming pulpal necrosis.

Figure 1(a) Initial panoramic radiograph. (b) Intra oral examination. (c) periapical radiograph. (d) CBCT, sagittal view. (e) CBCT, axial view.

**Figure:**
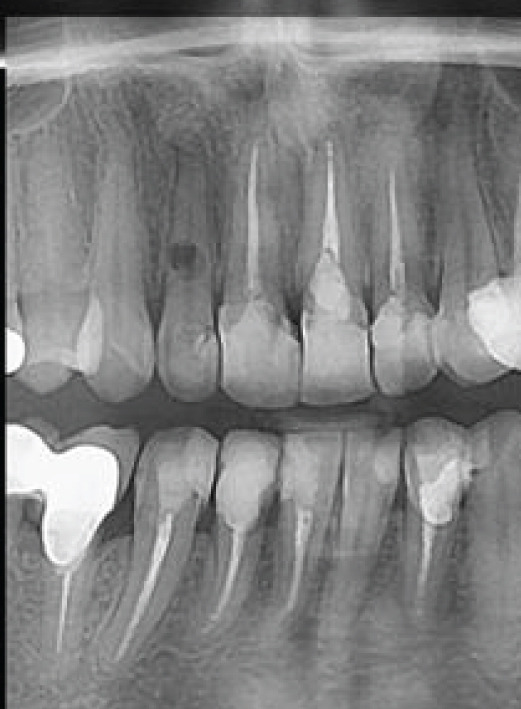
(a)

**Figure:**
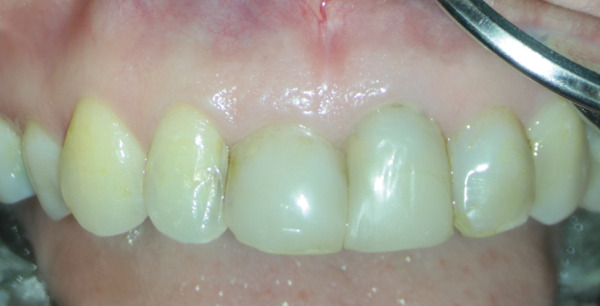
(b)

**Figure:**
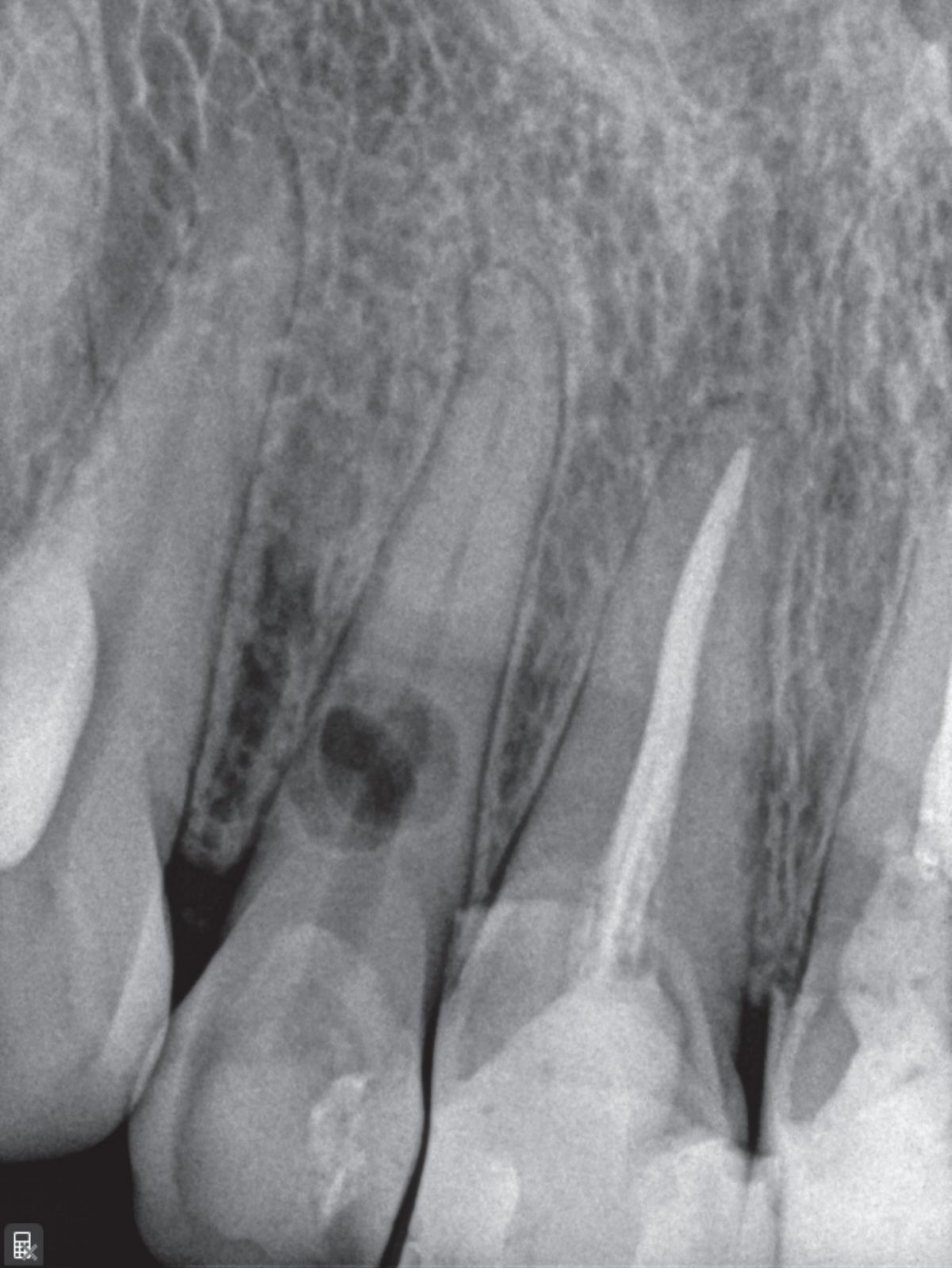
(c)

**Figure:**
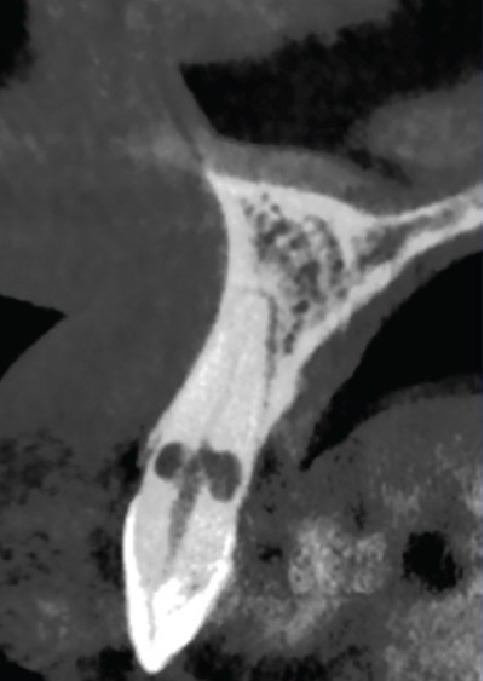
(d)

**Figure:**
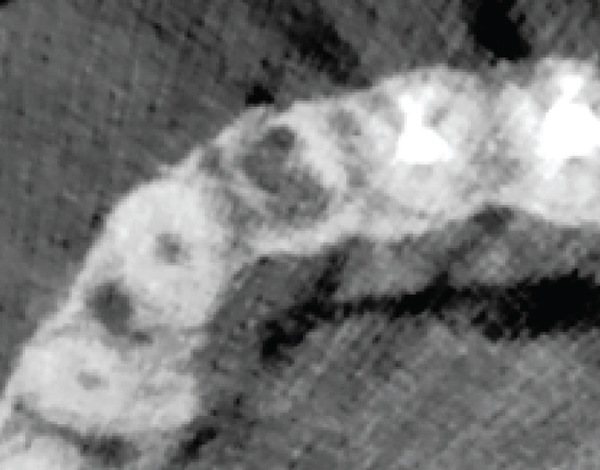
(e)

A periapical radiograph revealed two oval, balloon‐shaped radiolucencies with smooth borders in the coronal third of the root canal (Figure 1c). To further evaluate the defect, CBCT imaging was performed using a CS 8100 3D unit (Carestream Dental, Atlanta, GA, United States) with the following parameters: voxel size of 0.075 mm, FOV 5 × 5 cm, 90 kVp, 5 mA, exposure time of 10.8 s.

CBCT analysis revealed the following view‐specific findings:

• *Axial view*: A well‐circumscribed resorptive cavity with thinning of the buccal canal wall and loss of continuity at the coronal third.

• *Sagittal view*: A round radiolucent defect superimposed on the canal path, suggesting internal morphology without significant tunneling.

• *Coronal view*: Buccolingual thinning of dentin with a perforation on the buccal aspect; the canal outline became indistinct within the defect.

These features were most consistent with ECR, although a perforating IRR could not be fully excluded. The possible coexistence of IRR on the palatal aspect and ECR on the buccal aspect—eventually merging—was considered (Figure 1d,e; cross‐sectional CBCT view).

Treatment options were discussed, and nonsurgical root canal therapy was selected. After administration of local anesthesia (2% lidocaine with 1:100,000 epinephrine; Exir Pharmaceutical Co., Iran), an access cavity was prepared under rubber dam isolation. Cleaning and shaping were performed under a dental operating microscope (DOM; Magna, Labomed, United States) using a Neoniti A1 #20 rotary instrument (Neolix, France). Working length was established using an apex locator (Dempex, DEM Ltd., England) and confirmed radiographically (Figure 2a). Due to active intracanal bleeding, calcium hydroxide (Merck KGaA, Germany) was placed as an intracanal medicament, and the access cavity was sealed with IRM (Dentsply, Germany).

Figure 2(a) Working length determination; (b) sealed irrigation approach; (c) schematic illustration of the sealed irrigation setup; (d) obturation of the canal and resorptive defects; (e) final restoration with composite resin; (f) 3‐month follow‐up; and (g) 17‐month follow‐up.

**Figure:**
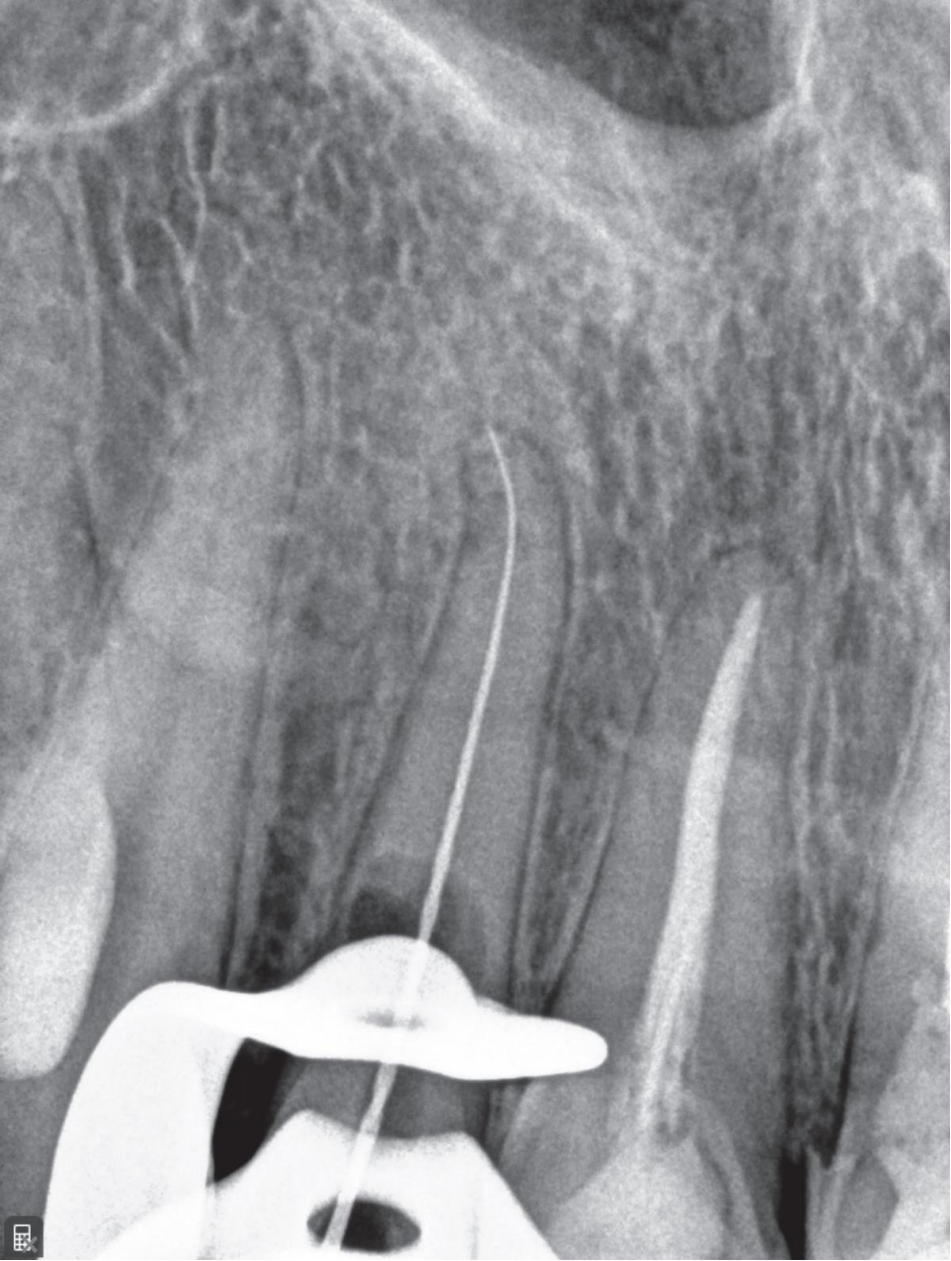
(a)

**Figure:**
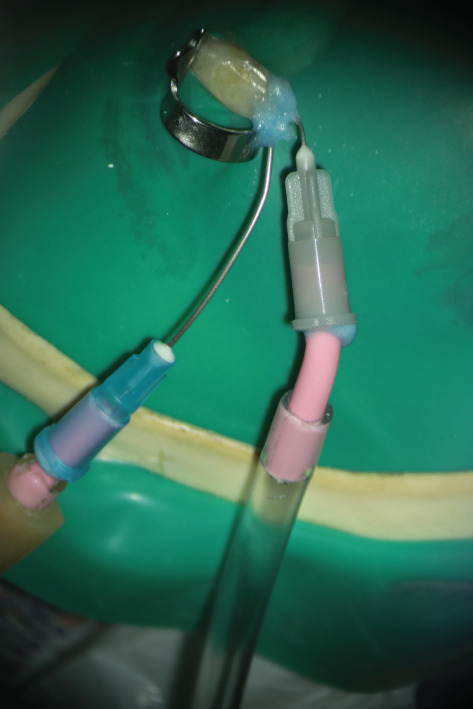
(b)

**Figure:**
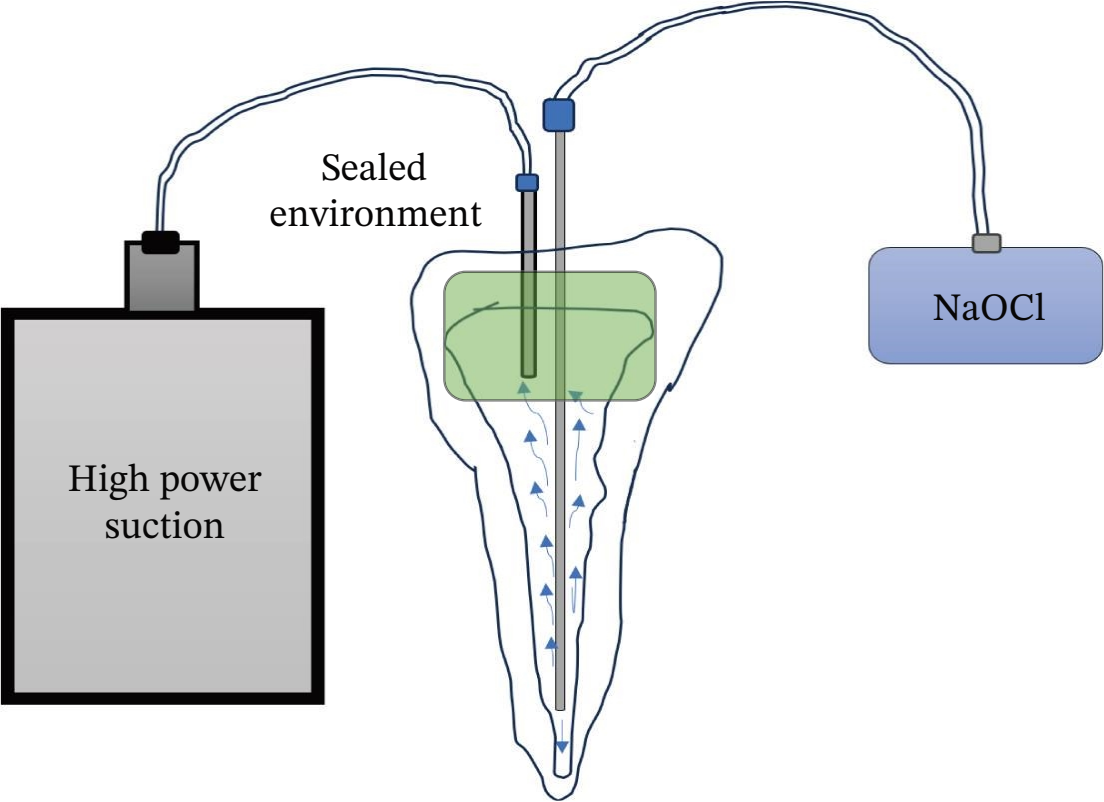
(c)

**Figure:**
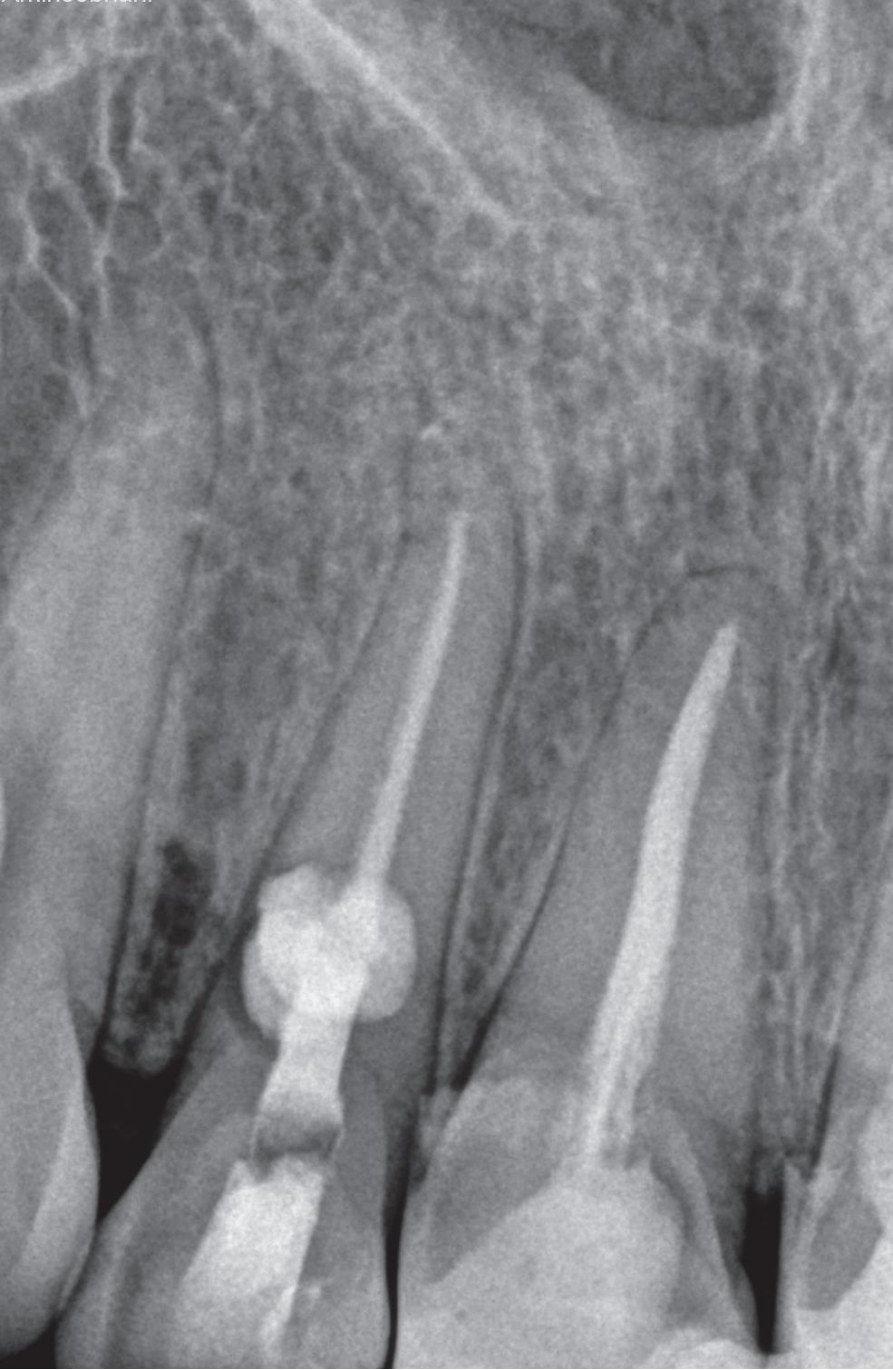
(d)

**Figure:**
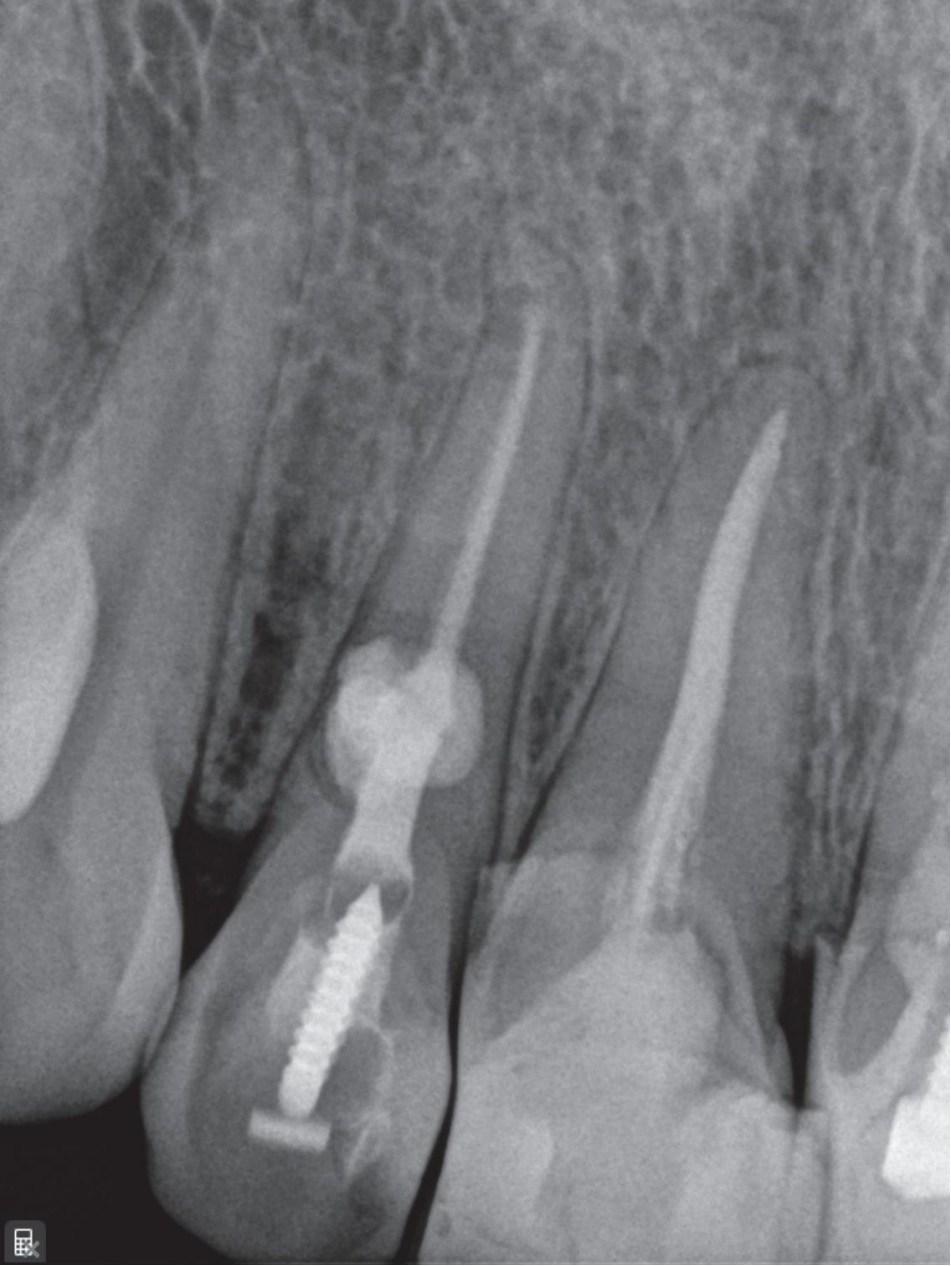
(e)

**Figure:**
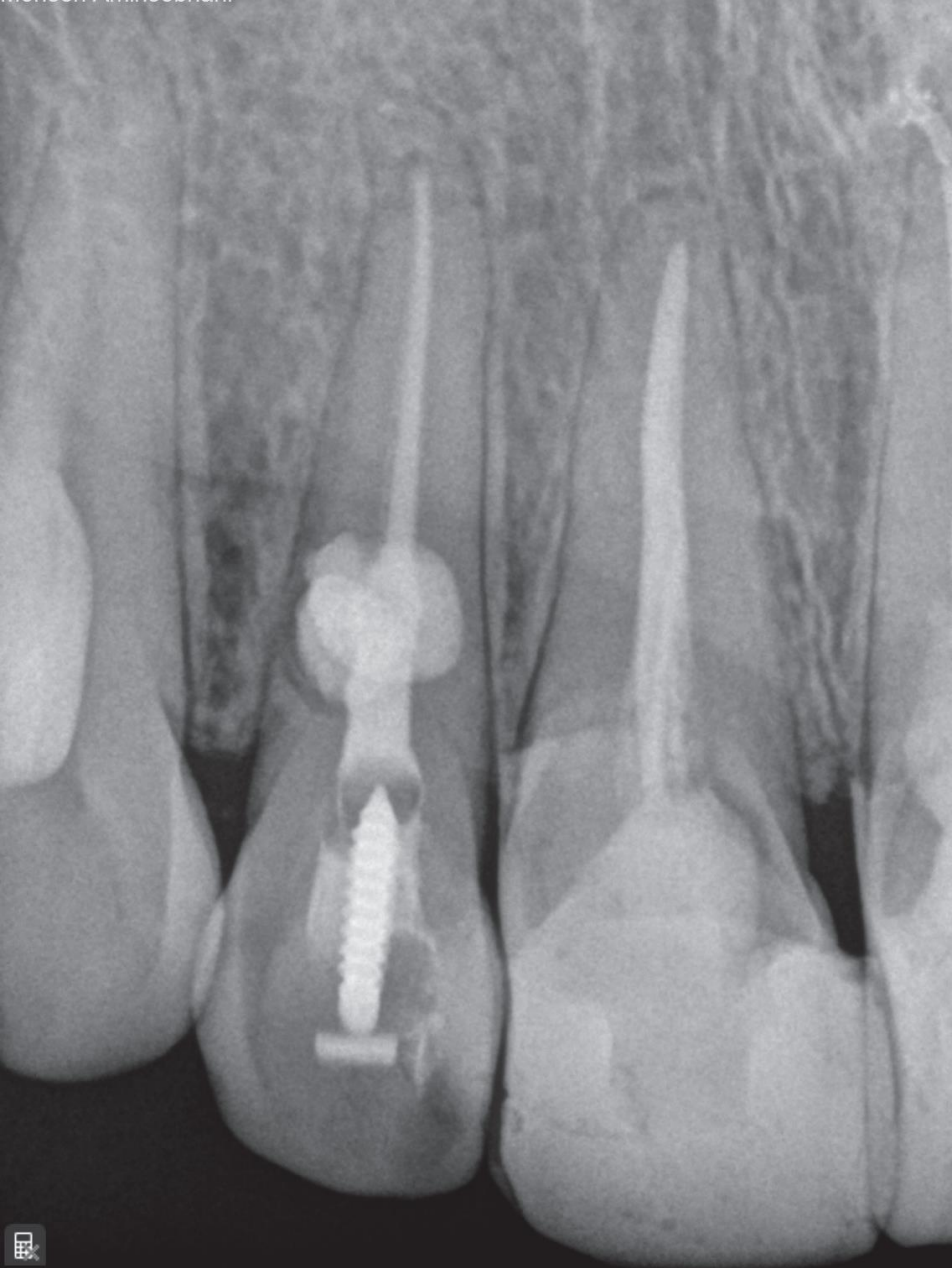
(f)

**Figure:**
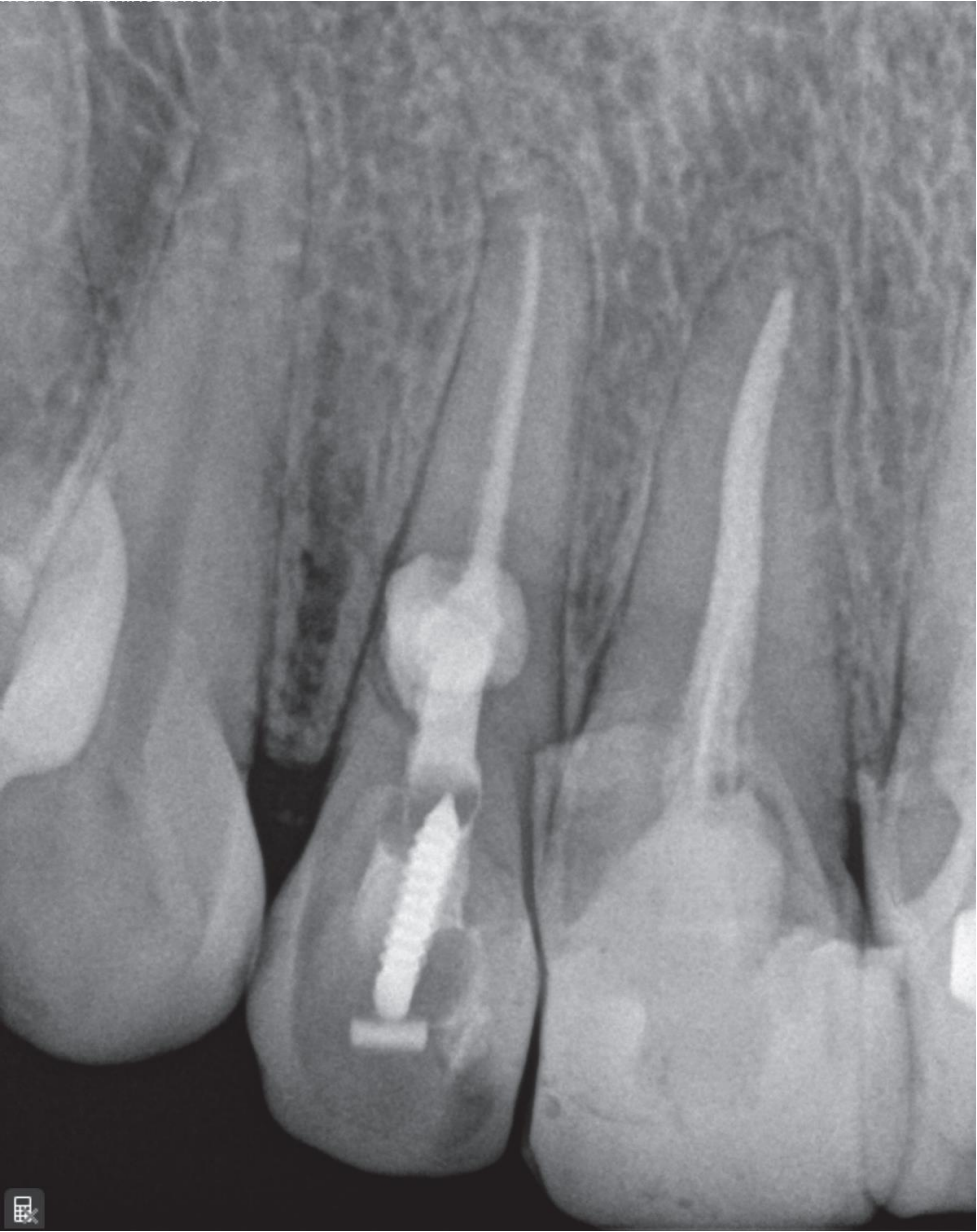
(g)

### 2.1. Second Visit (2 Weeks Later)

After removal of the temporary restoration and reisolation, the canal was refined with a #30 NiTi hand file (Mani Inc., Japan). A dual‐needle sealed irrigation system was used (Figure 2b,c). A 27‐gauge needle was positioned 2–3 mm short of the working length and connected to a reservoir of 5.25% NaOCl, whereas a 22‐gauge needle placed coronally was connected to high‐power suction (Techno‐Gaz, Italy). The access cavity and needle interface were sealed with a liquid dam (FlexDam, Turkey) to create a closed irrigation chamber. Continuous apical delivery and simultaneous coronal evacuation of NaOCl were performed for approximately 10 min, enabling safe irrigant exchange and effective debridement without extrusion.

Following irrigation, obturation was performed using an MTA‐based sealer (EdgeMTA, Hammers Dental, Iran). A gutta‐percha cone coated with sealer was placed to working length and trimmed at the cervical third (Figure 2d). The access cavity was temporarily restored, and the patient was referred for definitive coronal restoration (Figure 2e).

Detailed technical specifications of the sealed irrigation setup employed in this case are presented in Table 1.

**Table 1:** Sealed dual needle irrigation protocol.

Component	Specification/technique	Purpose/mechanism
Apical cannula	27‐gauge microcannula positioned 2–3 mm short of working length	Passive entry point for irrigant into the canal
Coronal suction cannula	22‐gauge suction needle connected to high‐vacuum unit	Primary driver for irrigant movement via coronal negative pressure
Seal of access	Light‐cured liquid dam sealing around both cannulas	Creates a closed system to maintain negative‐pressure dynamics
Irrigant	5.25% sodium hypochlorite (NaOCl)	Disinfection and tissue dissolution
Flow dynamics	Irrigant passively drawn coronally; no active apical delivery pressure	Prevents apical pressurization; enables controlled fluid movement
Pressure profile	No apical positive pressure applied at any stage	Eliminates risk of uncontrolled extrusion
Extrusion control	Any leakage limited to minimal controlled contact with the PDL space only	Safety in perforated canals; avoids spread to broader periapical tissues
Irrigation duration	Continuous irrigation for approximately 10 min	Ensures sustained irrigant exchange and cleaning

### 2.2. Follow‐Up

Clinical and radiographic evaluations were performed at 1‐, 3‐, 6‐, and 17‐month intervals. At the 17‐month follow‐up, the tooth remained asymptomatic and functional (Figure 2f,g). Radiographs demonstrated unchanged periapical bone structure, intact lamina dura, and no further progression or recurrence of resorption, indicating a favorable treatment outcome.

## 3. Discussion

Resorptive lesions are among the most challenging conditions to manage in endodontics. Their irregular, concave anatomy prevents instruments from contacting all affected surfaces, resulting in persistent infected tissue that may compromise treatment. Irrigation therefore becomes central to successful management, compensating for the limitations of mechanical debridement and enabling more complete disinfection [11].

A central challenge in this case was the diagnosis. The lesion exhibited several features typical of IRR, including a balloon‐like configuration, smooth borders, and a canal outline merging with the defect on CBCT. Clinically, the absence of probing defects, bleeding on probing, or visible cervical discoloration further supported IRR. However, the presence of a buccal perforation suggested an ECR, consistent with the ESE position statement indicating that IRR and ECR cannot always be differentiated with certainty [1]. Regardless of etiology, both forms create complex irregular spaces requiring enhanced irrigation strategies.

A key factor influencing treatment outcomes in resorptive lesions is the rate of lesion progression and the extent of structural damage. Slowly progressing IRR confined within the canal space generally has a favorable prognosis after adequate disinfection and obturation. In contrast, rapidly progressing or perforating IRR lesions carry a poorer prognosis due to greater destruction of dentin and potential communication with periodontal tissues [12]. Similarly, early‐stage ECR lesions tend to respond well to nonsurgical management, whereas advanced ECR with deep tunneling or pulpal involvement often requires surgical repair or even extraction [4]. In the present case, although perforation was present, the round, symmetrical internal morphology indicated a more controlled, slow‐progressing pattern, which may partly explain the favorable clinical outcome observed at long‐term follow‐up.

The concave surfaces and irregular extensions seen in resorptive defects significantly limit the effectiveness of conventional mechanical instrumentation, increasing the risk of residual infected tissue [12]. For this reason, irrigation strategies play a pivotal role in achieving a successful outcome [13].

Traditional positive‐pressure syringe irrigation is often inadequate for delivering irrigants into complex resorptive spaces and carries a documented risk of apical extrusion [14]. Although negative‐pressure systems such as EndoVac improve safety and irrigant retrieval [15, 16], they rely on passive apical inflow and may still fail to reach all extensions of IRR defects. Similarly, techniques such as PUI [17], EndoActivator [18], and GentleWave [19] enhance irrigant activation but do not consistently ensure complete penetration into large resorptive cavities.

The sealed irrigation technique used in this case was designed to address these limitations by combining targeted apical delivery and simultaneous coronal suction within a closed system. By placing a microcannula 2–3 mm short of the working length for controlled NaOCl penetration and using a coronally positioned macrocannula connected to high‐power suction, the system establishes a stable negative‐pressure flow environment. This design allows continuous irrigant exchange, minimizes apical pressurization, and enhances penetration into irregular resorptive anatomy while reducing the risk of extrusion.

Importantly, no positive pressure is applied apically in this technique. Irrigant movement is driven solely by coronal suction, generating a controlled negative‐pressure gradient similar to dual‐needle negative‐pressure designs, although the suction source is located coronally rather than apically. A comparable concept, termed the “Pulp Sucker,” was previously described by Buchanan and Verbanck in 2009 in a technical report shared online, though not formally published in peer‐reviewed literature (https://planbdental.com).

This approach differs fundamentally from traditional negative‐pressure systems such as EndoVac, which depend entirely on apical suction. In EndoVac, apical microcannula obstruction by debris can limit irrigant inflow, especially in large defects. In contrast, the apical delivery component in the present technique may enhance irrigant access to lateral extensions, undercuts, and perforating defects—features common in advanced IRR. This is particularly relevant in cases where the resorptive lesion communicates with periodontal tissues, as thorough debridement is essential to preventing reinfection and halting further progression.

The sealed irrigation environment also had a notable practical advantage in this patient, who reported discomfort related to the odor of sodium hypochlorite due to her asthma. The closed chamber prevented aerosolization and odor exposure, allowing safe use of NaOCl without respiratory irritation.

The absence of postoperative symptoms and the stable radiographic appearance at long‐term follow‐up support the effectiveness and safety of this approach. Nonetheless, potential limitations include technical sensitivity, difficulty in adapting the microcannula in narrow or curved canals, and the need for controlled isolation. Further clinical research is necessary to evaluate the reproducibility and wider applicability of this system.

A comparison of key features of positive‐pressure irrigation, negative‐pressure systems, and the sealed irrigation approach used in the present case is summarized in Table 2 [20–22].

**Table 2:** Comparison of positive pressure, negative pressure, and sealed irrigation techniques in root canal treatment.

Parameter	Positive pressure irrigation	Negative pressure irrigation	Sealed irrigation technique (this case)
Delivery method	Syringe and needle positive pressure.	Irrigant drawn apically via suction; no direct apical delivery.	Direct apical delivery via microcannula, combined with coronal suction.
Risk of apical extrusion	High, especially if needle binds or excessive pressure applied.	Very low; suction prevents extrusion.	Very low; sealed system and coronal suction control flow and pressure.
Irrigant penetration in complex anatomy	Often inadequate; limited to areas near the needle tip.	Improved, but relies on passive flow toward apex.	Enhanced; direct delivery into apical third may better penetrate irregularities.
Debris removal efficiency	Moderate; debris may remain in irregular spaces.	Good; improved evacuation of debris and irrigant.	Potentially superior; continuous exchange and sealed environment enhance debris removal.
Sealing of access cavity	Not sealed; open system.	Not sealed; open system.	Sealed access cavity maintains controlled irrigation environment.
Complexity of procedure	Low.	Moderate; requires specialized equipment and technique.	Moderate; requires specialized equipment and technique.
Cost	Low.	Moderate; commercial systems like EndoVac.	Low.
Evidence base	Extensive; limitations well documented.	Extensive; safety and efficacy established.	Limited; novel approach requiring further clinical validation.

In conclusion, this report describes the use of a sealed irrigation technique in the management of a resorptive lesion with features most consistent with IRR, though an ECR component could not be excluded. By combining apical delivery with coronal suction in a sealed system, this method addresses key limitations of both positive and negative pressure irrigation and offers a promising strategy for managing complex resorptive defects.

## Funding

No funding was received for this manuscript.

## Consent

Written informed consent was obtained from the patient for the publication of all clinical information, radiographs, and images included in this manuscript.

## Conflicts of Interest

The authors declare no conflicts of interest.

## Data Availability

The data supporting the findings of the present study will be provided by the corresponding author upon request.
